# Supplementary material for: ATG16L1 WD domain and linker regulates lipid trafficking to maintain plasma membrane integrity to limit influenza virus infection
Source: Autophagy. 2025 Apr 3;21(9):1911–26. doi: 10.1080/15548627.2025.2482516 (PMC12366830; doi:10.1080/15548627.2025.2482516)
Supplement: Figure_S1_R5.docx [file KAUP_A_2482516_SM0287.docx]

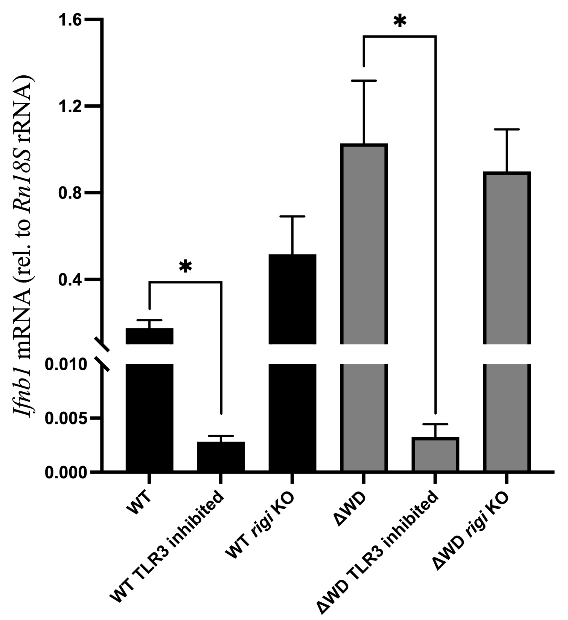

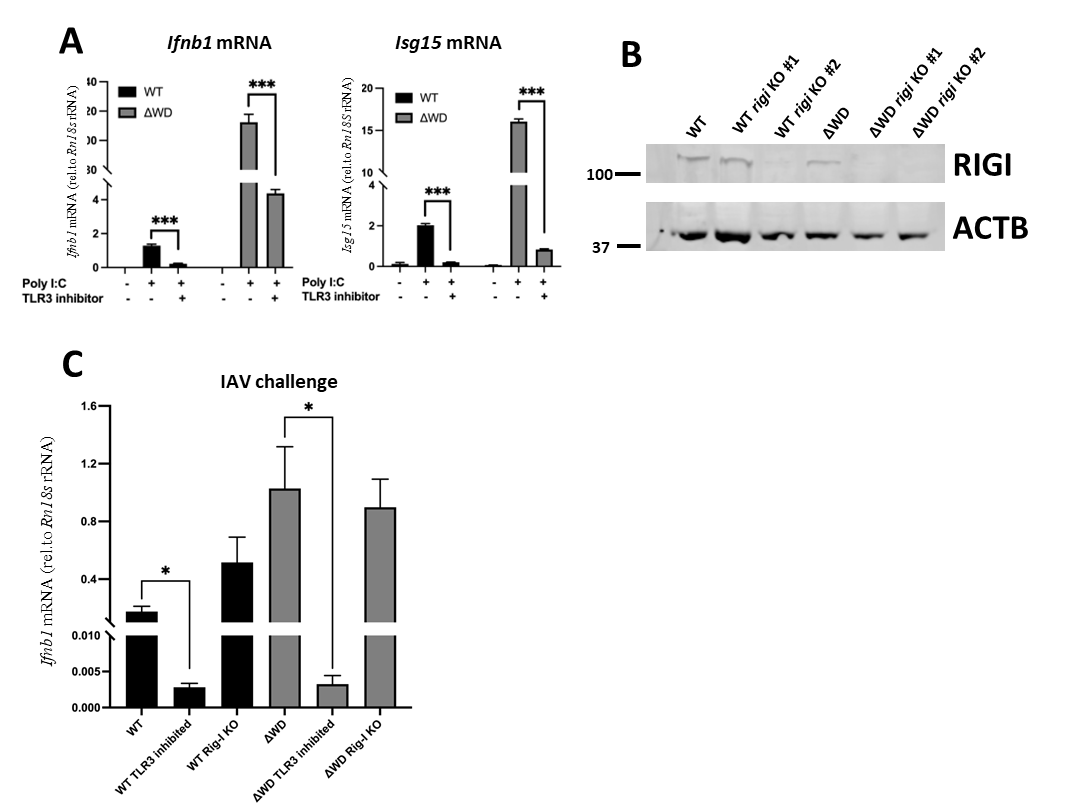


**Figure S1**. ATG16 L1 WD domains attenuated inflammatory signaling through the TLR3 pathway. (**A**) Expression of *Ifnb1* and *Isg15* mRNA relative to *Rn18s* rRNA in WT and ΔWD MEFs RNA (+SEM) by qPCR either untreated, or 4 h after incubation with poly IC, or treated with TLR3 inhibitor 1 h prior to incubation with poly IC. Independent samples t-test: *** = p<0.001. n=3. (**B**) *rigi* KO cell lines were generated by infecting WT and ΔWD MEFs with custom CRISPR gRNA lentivirus transduction particles. Knockdown of RIGI protein expression was evaluated by western blot. WT *rigi* KO #2 and ΔWD *rigi* KO #2 cell lines were chosen for IAV challenge. (**C**) *Ifnb1* mRNA expression (+SEM) following challenge with IAV for 120 min in WT and ΔWD cells with inhibition of either TLR3 or RIGI pathways. Independent samples t-test: * p<0.05 n=3.
